# The role of hemodialysis machines dedication in reducing Hepatitis C transmission in the dialysis setting in Iran: A multicenter prospective interventional study

**DOI:** 10.1186/1471-2369-5-13

**Published:** 2004-10-07

**Authors:** Alireza Abdollah Shamshirsaz, Mohammad Kamgar, Mir Reza Bekheirnia, Farzam Ayazi, Seyed Reza Hashemi, Navid Bouzari, Mohammad Reza Habibzadeh, Nima Pourzahedgilani, Varshasb Broumand, Amirhooshang Abdollah Shamshirsaz, Maziyar Moradi, Mehrdad Borghei, Niloofar Nobakht Haghighi, Behrooz Broumand

**Affiliations:** 1 Nephrology, Hazrat-e-Rasoul hospital, Tehran, Iran; 2 Nephrology, Pars Hospital, Tehran, Iran; 3 Academy of Medical Sciences, P.O.Box: 19395/4655, Tehran, Iran; 4 Research and Education Department, Charity Foundation for Special Diseases Tehran, Iran

## Abstract

**Background:**

Hepatitis C virus (HCV) infection is a significant problem among patients undergoing maintenance hemodialysis (HD). We conducted a prospective multi-center study to evaluate the effect of dialysis machine separation on the spread of HCV infection.

**Methods:**

Twelve randomly selected dialysis centers in Tehran, Iran were randomly divided into two groups; those using dedicated machines (D) for HCV infected individuals and those using non-dedicated HD machines (ND). 593 HD cases including 51 HCV positive (RT-PCR) cases and 542 HCV negative patients were enrolled in this study. The prevalence of HCV infection in the D group was 10.1% (range: 4.6%– 13.2%) and it was 7.1% (range: 4.2%–16.8%) in the ND group. During the study conduction 5 new HCV positive cases and 169 new HCV negative cases were added. In the D group, PCR positive patients were dialyzed on dedicated machines. In the ND group all patients shared the same machines.

**Results:**

In the first follow-up period, the incidence of HCV infection was 1.6% and 4.7% in the D and ND group respectively (p = 0.05). In the second follow-up period, the incidence of HCV infection was 1.3% in the D group and 5.7% in the ND group (p < 0.05).

**Conclusions:**

In this study the incidence of HCV in HD patients decreased by the use of dedicated HD machines for HCV infected patients. Additional studies may help to clarify the role of machine dedication in conjunction with application of universal precautions in reducing HCV transmission.

## Background

Hepatitis C virus (HCV) transmission occurs mainly through large or repeated direct percutaneous punctures to blood vessels; for example repeated injections for drug abuse [1]. Less frequent routes are sexual transmission [2], perinatal transmission [3], acquisition from mucous membrane exposure [4,5], body fluids [6] and colonoscopy [7]. However, in up to 40% of infected individuals, the route of transmission remains unknown [8]. Since the introduction of blood and organ donor screening by antibody testing in 1991, HCV has rarely been transmitted by transfusion of blood products[1], but there remains a relatively high incidence of new infections in hemodialysis (HD) units [9,10]. Several reports around the world indicate that the frequency of HCV is higher in patients undergoing maintenance HD than in the general population. The reported prevalence of HCV infection in maintenance HD patients varies markedly from country to country and from one center to another [11] ranging between 8% and 39% in North America, 1% and 54% in Europe, 17% and 51% in Asia, and 1% and 10% in Australia [12]. In Iran the prevalence of HCV varies from 5.5%–24%. [13,15].

Molecular virological studies have clearly shown the nosocomial transmission of HCV to hemodialysis patients,[16,17] but the exact modes of transmission remain unclear. Studies suggest several risk factors, including transmission through blood components [18]; patient-to-patient transmission through shared equipment [19], devices [20], or multidose vials [21]; and between patients treated on the same shift but not sharing equipment [16]. Basic hygienic precautions, for instance hand washing, the use of protective gloves when patients and HD equipment is touched are observed worldwide but only a few centers have isolated their HCV-positive patients or dialyzed them during dedicated shift or using dedicated dialysis machines. At the present time, the Center for Disease Control and Prevention (CDC) does not recommend isolation of patients with HCV [1]. The evaluation of this problem is difficult because of the paucity of prospective studies and the scarce data about patient-to-patient transmission in settings other than HD centers [6] and therefore the benefit of isolation of HCV infected dialysis patients remains controversial. The prevalence of HCV in hemodialysis units is higher than normal population in Iran (5–24% [13,15] versus 0.3 [22]) and most other countries. Considering the added expense of patient isolation we conducted a prospective study in hemodialysis units in Tehran, Iran, to evaluate the role of HD machine separation in reducing HCV transmission to HD patients.

## Methods

Among 40 HD centers in Tehran, we randomly selected centers one by one to reach a total number of 593 patients (12 centers) to enroll in this study. Selected centers were randomly divided in to dedicated (D) and non-dedicated (ND) HD machine groups, including 297 patients in D (4 centers) and 296 patients in ND group (8 centers). ELISA III checked all patients for HCV antibody detection before enrolling in the study. Positive cases were confirmed by RT-PCR. Only patients who were HCV positive by RT-PCR were considered to be HCV infected. Out of 593 HD cases 51 were RT-PCR positive (30 in the D groups and 21 in the ND group), and 542 were HCV negative (267 in the D group and 275 in the ND group). The prevalence of HCV infection in the D group was 10.1% (range: 4.6%– 13.2%) and was 7.1% (range: 4.2%–16.8%) in ND group. During the study conduction, 5 new HCV positive cases (1 in D group and 4 in ND group) and 169 new HCV negative cases entered the study. Information regarding age, sex, occupation (health care personnel, surgeons and dentists), HCV infected relatives, previous peritoneal dialysis, surgery during last 2 months, duration of hemodialysis, number of blood product transfusions, history of organ transplantation, and the causes of ESRD was collected. The obtained history of IV drug abuse, tattooing, and multiple sex partners was not reliable.

442 patients (254 cases in the D and 192 in the ND group) were followed for 9 months (first follow up population). 281 patients (160 cases in the D and 121 cases in the ND group) who remained within our study were followed for an additional 9 months (Second follow up population). Histories of surgeries or blood product transfusion were obtained at each follow-up; with no significant difference found between the D and the ND groups. There were no significant differences between the D and the ND groups in the number of patients lost to follow-up due to death, renal transplantation or transfer to a different hospital change (data not shown).

Patients were dialyzed for 4 or 4.5 hours, 2 or 3 times weekly, using standard HD techniques by Cuprophane and Polysulfone dialyzers. All included HD patients were HIV and HBs-Ag negative. Dialysis membranes were low-pressure and used only once and HD machines were bleached and rinsed between dialysis sessions according to the manufacturers' instruction. Socioeconomic level was essentially similar between D and ND groups. The only difference between two groups of HD centers was that in-group D, HCV positive patients were assigned to a dedicated HD machine, but in-group ND, HD HCV positive and negative patients were not assigned to dedicated machines. All machines were located in dialysis wards and not in separate rooms in both groups.

Patient to staff ratio in the D and the ND groups was not statistically different (3.1 and 3.4 respectively) and all staff members were negative for anti-HCV. To prevent HCV transmission, educational courses were held for the staff to reemphasize the CDC hygienic guidelines; however, an interview of all nurses directly involved in patient care disclosed some deviation from CDC hygienic guidelines. The minority of nurses remembered situations when they had failed to change their gloves due to an urgent adjustment of a hemodialysis machine. A checklist was used respecting hemodialysis-specific infection control practices and new gloves were applied for each individual patient. Nevertheless, masks, aprons and protective glasses were not universally used. In all centers, all patients had specific dialysis stations assigned to them, and chairs and beds were cleaned after each use. Handling and storage of medications and hand washing were not done in the same or adjacent areas to those where used equipment or blood samples were handled. One of the ND centers was excluded from the study due to non-adherence to CDC hygienic guidelines in the first months of the study.

Statistical analysis was performed using SPSS 10.5 software. Comparisons between groups were made by the chi-square test method for categorical variables and by the t-test for quantitative variables.

## Results

The mean age was 49.5 years (range from 12–84), 58.7% were male, and the mean HD duration was 21.6 months. The etiology of end-stage renal disease was hypertension in 36% followed by diabetes in 28% and glomerulonephritis in 10.5%. 15.5% of patients in the dialysis centers had a one-time history of kidney transplantation, and 2.2% had undergone transplantation twice. The demographic data for two groups is illustrated in table 1.

**Table 1:** Demographic characteristics of dedicated and non-dedicated groups.

	**Cases included at the beginning the study**		**Cases included during the study (new cases)**	
	**Dedicated**	**Non-dedicated**	**Dedicated**	**Non-dedicated**
**Total count of included cases**	267	275	85	84
**Age [Mean (SE)]**	48.5 (0.9)	50.6 (1.0)	47.9(3.1)	51.9(1.8)
**Male proportion (%)**	59.9	54.2	62.2	61.9
**At-risk occupation (%)**	0.4*	2.6	5.1	6.1
**Duration of HD [Mean (SE)]**	24.9(2.8)	25.2 (4.9)	12.6(4.8)	11.8(5.8)
**Previous peritoneal dialysis (%)**	6.9**	2.2	2.4	2.4
IV drug abuse (%)***	0.0	1.3	3.9	1.2
**Surgery during the last 2 months (%)**	1.9	1.5	12.2	8.3
**Transfusion during the last 2 months (%)**	27.0	21.0	22.0	19.0
**Previous transplantation (%)**	17.7	18.2	9.5	8.3

* P = 0.04 (Significant difference with the control group)

** P = 0.009 (Significant difference with the control group)

*** History of IV drug abuse was not ascertained.

All other differences between the D and the ND group were not significant.

In the first follow-up period, the incidence of HCV infection was 1.6% and 4.7% in the dedicated and the non-dedicated groups (p = 0.05). In the second follow-up period, the incidence was 1.3% in the dedicated and 5.7% in the non-dedicated groups (p < 0.05) (table 2).

**Table 2:** Incidence of HCV positive (PCR) cases in dedicated and non-dedicated groups in the first and second follow-up.

	**First follow-up**		**Second follow-up**	
	**Positive No (%)**	**Negative No (%)**	**Positive No (%)**	**Negative No (%)**
**Dedicated**	4(1.6)	250(98.4)	2 (1.3)	158 (98.7)
**Non-dedicated**	9(4.7)	183(95.3)	7 (5.8)	114 (94.2)
**P value**	= 0.05		<0.05	

## Discussion

The possibility of an intradialytic spread of HCV appeared to be very low and the treatment of HCV infected patients with dedicated machines was not strictly required [23-26]. Although there is no consensus regarding machine dedication between HCV non-infected and HCV infected patients, we found that using dedicated HD machines in both follow-up periods has an important role in reducing HCV transmission. Similar results have been shown previously. Low prevalence of HCV infection (HCV antibodies) in a HD unit in Istanbul (4.7%) showed that patient isolation and use of dedicated dialysis machines for seropositive patients decrease the transmission of HCV infection in HD centers [27]. Data derived from another study in Turkey demonstrated that nosocomial spread of HCV in HD units in which both seropositive and seronegative patients were treated together were higher than that of units with dedicated machines [28]. A study in Lebanon has shown that infection by HCV may be dialysis machine-related, rather than transfusion-related [29]. Another study from Portugal also demonstrated that the incidence of HCV infection was lowest in units that used dedicated machines or dedicated rooms for anti-HCV-positive patients [30]. Genotyping analysis in a molecular study confirmed that implementation of rigorous hygienic routines and introduction of dedicated rooms and machines for HCV-infected patients are important measures for effective control of HCV infection in a hemodialysis environment [31]. Findings from a study conducted in Shiraz, Iran, where 5.5% of patients were anti-HCV positive indicate that cross-infection by dialysis machines was mainly responsible for HCV infection. This study also reemphasized that cross infection through dialysis machines, rather than transfusion of blood products was the priming mode of transmission of hepatitis C virus among HD patients [15].

Some authors recommended that it is sufficient to treat every dialysis patient as potentially infectious, strictly adhering to the "universal precautions for prevention of HCV transmission", to prevent the spread of HCV in dialysis units [32,33] and isolation of HCV-infected dialysis patients and use of dedicated machines are unjustified [34]. P. Gilli et al demonstrated that machine separation in the presence of strict application of hygienic precautions did not reduce HCV transmission [35]. In agreement to this report, simpler measures such as the observance of Universal Precautions (UP), continuous training of the care staff and the use of anti-HCV positive patients personal instruments which can stop the diffusion of HCV infection in HD centers [36] have been mentioned.

In our study population, the prevalence of HCV infection was approximately the same in both groups at the beginning of the study, but significantly lower incidence of HCV infection in D group may show that machine dedication strategies can be effective to reduce HCV transmission at least in our HD centers.

## Conclusions

Considering the prevalence of HCV infection and adherence to adequate infection control measures, HD machine dedication may help to decrease transmission of HCV infection in our dialysis units.

However rigorous implementation of precaution measures remains a cornerstone for prevention of HCV transmission among patients undergoing maintenance hemodialysis, but as unpredictable accidents can always take place in hemodialysis units; machine dedication may play a more important role in prevention of HCV transmission. Further studies are needed to evaluate the possible roles of machine dedication in the presence of strict adherence to hygienic precautions.

## Authors' contributions

Dr AA Shamshirsaz designed the draft questionnaires and study protocol, managed the coordination of the surveys and drafted the manuscript. . Dr. Kamgar participated in drafting the manuscript and data collection, coordinated the study and designed the protocol. Dr Bekheirnia conceived of and designed the study, performed the statistical analysis, drafted the manuscript and helped design the protocol. Dr Bouzari helped draft the manuscript, participated in statistical analysis and managed paraclinical surveys. Dr Habibzadeh drafts the manuscript, participated in statistical analysis and helped in data collection and physical examination. Dr Pourzahedgilani participated in physical exam, filled out questionnaires and searched scientific sources. Dr V Broumand participated in drafting the manuscript. Dr Moradi participated in statistical analysis. Dr. Ayazi, Dr. Hashemi, Dr. A.H Shamshirsaz, Dr. Borghei and Dr. Haghighi took part in physical exams and filling questionnaires. Dr. B Broumand facilitated the study design and progress.

## Pre-publication history

The pre-publication history for this paper can be accessed here:


